# *In vitro* Activity of Antivirulence Drugs Targeting the *las* or *pqs* Quorum Sensing Against Cystic Fibrosis *Pseudomonas aeruginosa* Isolates

**DOI:** 10.3389/fmicb.2022.845231

**Published:** 2022-04-25

**Authors:** Diletta Collalto, Giulia Giallonardi, Alessandra Fortuna, Carlo Meneghini, Ersilia Fiscarelli, Paolo Visca, Francesco Imperi, Giordano Rampioni, Livia Leoni

**Affiliations:** ^1^Department of Science, Roma Tre University, Rome, Italy; ^2^Wellcome Centre for Integrative Parasitology, Institute of Infection, Immunity and Inflammation, University of Glasgow, Glasgow, United Kingdom; ^3^Laboratory of Cystic Fibrosis Microbiology, Diagnostic Medicine and Laboratory, Bambino Gesú Hospital, Rome, Italy; ^4^Santa Lucia Foundation (IRCCS), Rome, Italy

**Keywords:** *Pseudomonas aeruginosa*, cystic fibrosis, antivirulence drugs, quorum sensing, quorum quenching, clofoctol, niclosamide

## Abstract

The chronic lung infection caused by *Pseudomonas aeruginosa* is a major cause of morbidity and mortality in cystic fibrosis (CF) patients. Antivirulence drugs targeting *P. aeruginosa* quorum sensing (QS) systems are intensively studied as antibiotics substitutes or adjuvants. Previous studies, carried out in non-CF *P. aeruginosa* reference strains, showed that the old drugs niclosamide and clofoctol could be successfully repurposed as antivirulence drugs targeting the *las* and *pqs* QS systems, respectively. However, frequent emergence of QS-defective mutants in the CF lung undermines the use of QS inhibitors in CF therapy. Here, QS signal production and susceptibility to niclosamide and clofoctol have been investigated in 100 *P. aeruginosa* CF isolates, with the aim of broadening current knowledge on the potential of anti-QS compounds in CF therapy. Results showed that 85, 78, and 69% of the CF isolates from our collection were proficient for the *pqs*, *rhl*, and *las* QS systems, respectively. The ability of both niclosamide and clofoctol to inhibit QS and virulence *in vitro* was highly variable and strain-dependent. Niclosamide showed an overall low range of activity and its negative effect on *las* signal production did not correlate with a decreased production of virulence factors. On the other hand, clofoctol displayed a broader QS inhibitory effect in CF isolates, with consequent reduction of the *pqs*-controlled virulence factor pyocyanin. Overall, this study highlights the importance of testing new antivirulence drugs against large panels of *P. aeruginosa* CF clinical isolates before proceeding to further pre-clinical studies and corroborates previous evidence that strains naturally resistant to QS inhibitors occur among CF isolates. However, it is also shown that resistance to *pqs* inhibitors is less frequent than resistance to *las* inhibitors, thus supporting the development of *pqs* inhibitors for antivirulence therapy in CF.

## Introduction

The opportunistic human pathogen *Pseudomonas aeruginosa* can cause a variety of different acute and chronic infections that are hard to eradicate due to the ability of this bacterium to form biofilms, resist to available antibiotics, and acquire new resistance genes *via* horizontal gene transfer (Malhotra et al., 2019). *P. aeruginosa* is indeed included into the ESKAPE group of pathogens for which new antimicrobials are urgently needed (*Enterococcus faecium*, *Staphylococcus aureus*, *Klebsiella pneumoniae*, *Acinetobacter baumannii*, *P. aeruginosa*, *Enterobacter spp.*) (Rice, 2008; Boucher et al., 2009).

The research for new drugs to combat *P. aeruginosa* infections is a very active field where traditional antibiotic development comes together with novel strategies, including the development of antivirulence agents to be used alone or in combination with antibiotics. By targeting virulence determinants, antivirulence drugs disarm pathogens making them more vulnerable to the host immune system attack. In addition, since antivirulence drugs target non-essential functions; it is generally believed that they should impose a lower selective pressure for the emergence of resistance, compared with antibiotics (Dickey et al., 2017; Ellermann and Sperandio, 2020).

In *P. aeruginosa*, virulence genes expression is largely dependent on three quorum sensing (QS) systems. The *las* and *rhl* systems are based on *N*-3-oxododecanoyl-L-homoserine lactone (3OC_12_-HSL) and *N*-butanoyl-homoserine lactone (C_4_-HSL) as signal molecules, respectively, while the *pqs* system relies on signal molecules belonging to the 2-alkyl-4-quinolones (AQs) class, *i.e.*, 2-heptyl-4-quinolone (HHQ) and 2-heptyl-3-hydroxy-4-quinolone (PQS) (Williams and Càmara, 2009; Papenfort and Bassler, 2016). In the *P. aeruginosa* reference strains PAO1 or PA14 the *las* system is hierarchically dominant over the *rhl* and *pqs* systems. When 3OC_12_-HSL levels reach the *quorum* concentration, this molecule binds to the LasR signal receptor, which in turn activates the transcription of the cognate signal synthase gene *lasI*, causing a further increase in signal production. This autoinduction process is common to most QS systems, ensuring QS response robustness. Besides *lasI*, the LasR-3OC_12_-HSL complex induces the transcription of many genes, including those involved in the synthesis and reception of C_4_-HSL and AQs. Also in this case, the signal receptors RhlR and PqsR, upon activation by the cognate signal molecules C_4_-HSL and AQs, trigger the autoinduction process and promote the expression of additional target genes. Overall, the three QS systems of *P. aeruginosa* control hundreds of genes, including virulence and biofilm genes (Williams and Càmara, 2009; Papenfort and Bassler, 2016). Since *P. aeruginosa* mutants inactivated in each one of the three QS systems are significantly attenuated in animal and plant infection models, these systems are considered as good targets for the development of antivirulence drugs (Rampioni et al., 2014; Ellermann and Sperandio, 2020).

By combining target-oriented screening and drug-repurposing approaches, our research group has previously identified anti-QS secondary activities against *P. aeruginosa* in drugs already approved for use in humans (Imperi et al., 2013, 2019; D’Angelo et al., 2018; Mellini et al., 2019; Baldelli et al., 2020). Among these drugs, niclosamide and clofoctol are the most interesting ones due to their high anti-QS activity and low cytotoxicity (Imperi et al., 2013; Costabile et al., 2015; Rampioni et al., 2017a; D’Angelo et al., 2018).

The salicylanilide compound niclosamide has been developed for the treatment of tapeworm infections. Its primary mechanism of action is largely unknown, though it apparently involves uncoupling oxidative phosphorylation in adult tapeworms (Pearson and Hewlett, 1985). As an antivirulence drug, niclosamide targets the 3OC_12_-HSL signaling process by a still uncharacterized mechanism. In the reference strain *P. aeruginosa* PA14, niclosamide decreases the production of 3OC_12_-HSL and of QS-dependent virulence factors, such as pyocyanin and elastase, with consequent attenuation of virulence in the *Galleria mellonella* infection model (Imperi et al., 2013).

Clofoctol is a synthetic antibiotic active against numerous Gram-positive bacteria. With a few exceptions (*e.g.*, *Haemophilus influenzae*, *Bordetella spp*., *Neisseria meningitidis*, and *Neisseria gonorrhoeae*), clofoctol does not inhibit the growth of Gram-negative bacteria, including *P. aeruginosa* (Simonnet et al., 1979; Buogo, 1981, 1984). The mechanism of action of clofoctol is not yet fully elucidated, though it seems to be related to ATP synthesis inhibition, resulting in disruption of anabolic processes (Yablonsky, 1983; Bailly and Vergoten, 2021). As an antivirulence drug, clofoctol acts as a competitive inhibitor of the AQs signal receptor PqsR. Clofoctol-mediated PqsR inhibition leads to decreased production of AQs and hence of AQs-dependent virulence factors, with consequent attenuation of *P. aeruginosa* infectivity in *G. mellonella* (D’Angelo et al., 2018). Notably, both niclosamide and clofoctol disclose anti-biofilm activity (Imperi et al., 2013; D’Angelo et al., 2018).

A clinical context in which *P. aeruginosa* infections are very relevant is cystic fibrosis (CF), a genetic disease affecting about 70,000 people worldwide. About three-quarters of CF adults are chronically infected with *P. aeruginosa* (Malhotra et al., 2019). After the first isolation of *P. aeruginosa* in the lungs, usually during the pediatric age, a period of intermittent colonization of the airways begins, followed by the onset of a chronic infection. Early aggressive antibiotic therapies delay the onset of the chronic infection increasing life expectancy. However, once established, the chronic infection can be kept under control but cannot be eradicated by antibiotics, thus persisting in the CF patient lungs even for decades, causing inflammation and progressive loss of pulmonary function (Folkesson et al., 2012; Malhotra et al., 2019; Rossi et al., 2021). As a consequence of the patient-specific and years-long evolution within the lung, *P. aeruginosa* strains isolated from CF patients disclose genotypic and phenotypic variability (Rossi et al., 2021). Common phenotypes of *P. aeruginosa* strains from CF patients are biofilm overproduction, increased resistance to antibiotics, reduced motility and loss or attenuation of other virulence-related traits (Folkesson et al., 2012; Marvig et al., 2015; Winstanley et al., 2016).

Some phenotypes frequent in CF strains are accompanied by mutations in the *lasR* gene, coding for the signal receptor of the *las* QS system (Smith et al., 2006; Hoffman et al., 2009; Feltner et al., 2016). In fact, genomic studies identified a high frequency of mutations in the *lasR* gene in strains isolated from CF patients with chronic infection for more than a decade (Smith et al., 2006; Bjarnsholt et al., 2010). Strains impaired in the *rhl* or *pqs* system have also been isolated from CF patients, even if with lower frequency with respect to *las-*deficient strains (Bjarnsholt et al., 2010; Jiricny et al., 2014). For this reason, the importance of QS in CF chronic infection and the therapeutic potential of anti-QS drugs are still under debate (García-Contreras et al., 2015; García-Contreras, 2016). In this context, it should be emphasized that the vast majority of the anti-QS compounds identified have most often been tested only against few non-CF reference strains of *P. aeruginosa*, commonly shared by many laboratories worldwide (*e.g.*, PAO1 or PA14) or, at best, against a limited number of CF strains (García-Contreras et al., 2015; D’Angelo et al., 2018; Baldelli et al., 2020; Mahan et al., 2020).

On this basis, the objective of this study was to investigate the suitability of antivirulence drugs targeting the *las* or the *pqs* QS system for the treatment of CF patients infected with *P. aeruginosa*. For this purpose, 100 isolates from CF patients with intermittent or chronic lung infection were preliminarily characterized for their ability to produce QS signal molecules. To investigate if the activity disclosed by an anti-QS drug against a reference strain (non-CF) could be conserved against CF isolates, niclosamide and clofoctol were used as model compounds active against the *las* or *pqs* QS system, respectively.

Overall, our results show that resistance to anti-*las* and anti-*pqs* drugs naturally occurs in CF isolates, although less frequently for the latter drug. Our findings highlight the importance of testing new antivirulence drugs in large collections of strains at the early stages of their discovery, and support the development of anti-QS drugs targeting the *pqs* system for future applications in CF therapy.

## Materials and Methods

### Bacterial Strains, Media, and Chemicals

The bacterial strains and clinical isolates used in this study are listed in Supplementary Tables 1, 2, respectively. The strains were routinely grown at 37°C with aeration in Luria-Bertani broth (LB) supplemented, when required, with 3-(*N*-morpholino)propane sulfonic acid (MOPS; pH 7.0) at the final concentration of 50 mM. Synthetic 3OC_12_-HSL was prepared at the concentration of 10 mM in ethyl acetate acidified with 0.1% (v/v) acetic acid, while synthetic PQS stock solution was prepared at the concentration of 20 mM in methanol. Synthetic QS signal molecules were kindly provided by Proff. Paul Williams and Miguel Camara (Centre for Molecular Sciences, University of Nottingham, United Kingdom). Niclosamide and clofoctol were purchased from Sigma-Aldrich and dissolved in dimethyl sulfoxide (DMSO) at 10 and 80 mM final concentration, respectively.

### Detection and Quantification of Quorum Sensing Signal Molecules

Levels of 3OC_12_-HSL and AQ signal molecules were determined in culture supernatants of *P*. *aeruginosa* laboratory strains using the PA14-R3 (3OC_12_-Rep) and PAO1Δ*pqsA* P*pqsA*:*lux* (AQ-Rep) reporter strains (Supplementary Table 1), respectively, according to previously described procedures (Massai et al., 2011; D’Angelo et al., 2018). Briefly, bacterial cultures were grown in 96-well microtiter plates at 37°C with shaking. Supernatants were collected at the end of the exponential growth phase for 3OC_12_-HSL quantification or at the stationary phase for AQ quantification. Ten-μL of cell-free culture supernatants were added to 190 μL of LB MOPS inoculated with the 3OC_12_-Rep biosensor (final OD_600_ = 0.045) or 5 μL of cell-free culture supernatant were added to 195 μL of LB inoculated with the AQ-Rep biosensor (final OD_600_ = 0.1) in 96-wells black clear-bottom microtiter plates. Microtiter plates were incubated at 37°C with gentle shaking (120 rpm). The optical density at 600 nm wavelength (OD_600_) and relative light units (RLU) values were measured after 4-h or 6-h incubation for 3OC_12_-HSL or AQ measurements, respectively, by using an automated luminometer-spectrophotometer plate reader (TECAN Spark10M). Dedicated calibration curves were generated by growing each reporter strain in the presence of increasing concentrations of synthetic 3OC_12_-HSL or PQS. The resulting dose-response curves were used to calculate the concentration of each signal molecule in culture supernatants.

The C_4_-Rep biosensor (Supplementary Table 1) was used to detect the C_4_-HSL production in co-culture with *P. aeruginosa* CF strains, as previously described (Imperi et al., 2013). The C_4_-Rep reporter and each CF isolates were independently grown overnight at 37°C and co-inoculated in LB MOPS to an OD_600_ of 0.045 and 0.015, respectively. Aliquots (200 μL) of the co-culture were grown at 37°C in 96-wells black clear-bottom microtiter plates. The OD_600_ and RLU values were measured at the beginning of the stationary phase of growth by using an automated luminometer-spectrophotometer plate reader (TECAN Spark10M). The reporter activity was determined as the RLU/OD_600_ for each sample. Light emission of the PAO1/C_4_-Rep co-culture was used as positive control. A PAO1-derivative double mutant Δ*lasI*Δ*rhlI*, unable to produce 3OC_12_-HSL or C_4_-HSL, was used as a negative control (C_4_-HSL non-producer strain; Supplementary Table 1).

### Pyocyanin Production and Elastase Activity Assays

Pyocyanin was extracted and quantified as previously described (Essar et al., 1990) with minor modifications (Baldelli et al., 2020). Briefly, each CF isolate was incubated in 96-well microtiter plates at 37°C with gentle shaking (120 rpm) in 200 μL of LB broth supplemented with the antivirulence drug (20 μM niclosamide or 100 μM clofoctol) or DMSO (untreated control). After incubation, two independent cultures of the same strain were pooled, the OD_600_ was measured, and cell-free supernatants were collected into 1.5 mL tubes. After extraction with an isovolume of chloroform, the pyocyanin-containing chloroform phase was transferred into clean 1.5 mL tubes and acidified with an isovolume of 0.2 N HCl. After centrifugation, 200 μL of the aqueous-phase were transferred into 96-wells microtiter plates, and the amount of extracted pyocyanin was measured at an absorbance of 520 nm (A_520_) by using an automated plate reader (TECAN Spark10M).

Elastase activity was determined as reported in Ohman et al. (1980) with a few adjustments. Briefly, each CF isolate was incubated in 96-well microtiter plates for 24 h at 37°C with gentle shaking (120 rpm) in 200 μL of LB broth supplemented with 50 mM MOPS and the antivirulence drug (20 μM niclosamide) or DMSO (untreated control). After the incubation time, two independent cultures of the same isolate were pooled, the OD_600_ was measured and 40 μL of cell-free supernatants were added into 1.5 mL tubes containing the elastin-Congo red reaction buffer. Finally, the elastolytic activity was measured at A_495_ by using an automated plate reader (TECAN Spark10M).

The average measurements and relative standard deviations (SD) were calculated from at least three independent experiments.

### Statistical Analysis

Statistical analysis was performed by using the software GraphPad Prism (v. 6.01). The data distributions departed from a normal distribution therefore differences were statistically tested using the non-parametric Kolmogorov-Smirnov test (KS-test). The correlation among the parameters were statistically tested using the Pearson correlation. The *p*-values less than 0.05 were considered statistically significant.

## Results

### Production of Quorum Sensing Signal Molecules by *Pseudomonas aeruginosa* Isolates From Cystic Fibrosis Patients

The *P. aeruginosa* isolates tested in this study were isolated from the airways CF patients for up to 15 years from the establishment of the first pulmonary infection, and previously characterized for their antibiotic susceptibility profile (Imperi et al., 2019). In this study, the collection (100 isolates) has been grouped as follow: 40 isolates from the first documented infection (First isolate, F); 25 isolates from patients infected for 2–3 years (chronic Early, E); 25 isolates from patients infected for 4–7 years (chronic Middle, M); 10 isolates from patients infected for more than 15 years (chronic Late, L) (Supplementary Table 2). In addition, according to the European Centre for Disease Control (ECDC) criteria, the collection contains: 48 isolates susceptible to all antibiotics (Susceptible, S); 32 isolates not susceptible to antibiotics belonging to one or two different classes (Resistant, R); 20 isolates not susceptible to one or more antibiotics belonging to at least 3 different classes (Multi-Drug Resistant, MDR) (Supplementary Table 2).

The distribution of functional *las* and *pqs* QS systems in the collection was investigated by measuring the levels of 3OC_12_-HSL and AQs in the cell-free supernatants of each CF isolate, grown under standard laboratory conditions. Briefly, cell-free supernatants were used to induce bioluminescence emission by biosensors specific for 3OC_12_-HSL or AQs, and the concentration of signal molecule in each sample was determined by using calibration curves obtained with synthetic 3OC_12_-HSL or AQs (details in Materials and Methods). 3OC_12_-HSL and AQs levels ranged from undetectable to about 15 and 50 μM, respectively, while their concentration was ca. 3 and 15 μM, respectively, in the PAO1 reference strain (Supplementary Figure 1).

**FIGURE 1:**
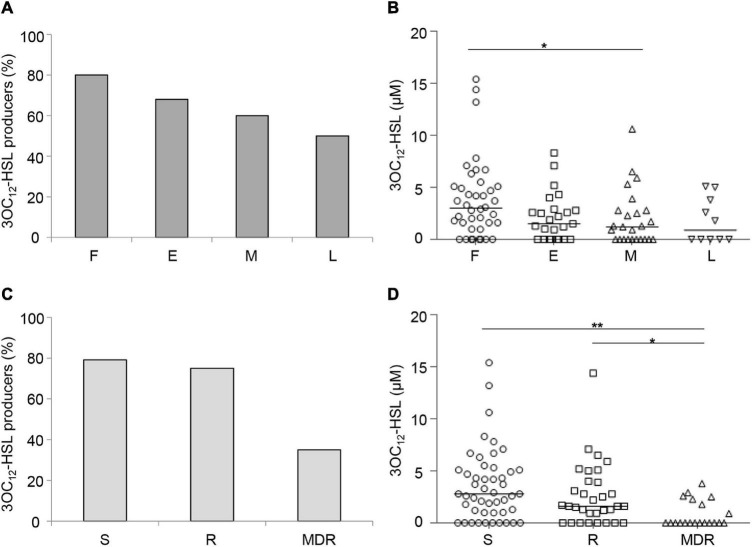
Production of 3OC_12_-HSL by CF isolates. Percentage of QS signal molecule-producing isolates grouped by duration of the chronic lung infection **(A)** or by antibiotic susceptibility pattern **(C)**. 3OC_12_-HSL levels produced by CF isolates grouped by the duration of the chronic lung infection **(B)** or by the antibiotic susceptibility pattern **(D)**. Horizontal lines are the median value. Asterisks denote statistically significant differences between groups (*p < 0.05; **p < 0.01; KS-test). F, first isolate; E, chronic early; M, chronic middle; L, chronic late; S, susceptible to all antibiotic classes; R, non-susceptible to one or two classes of antibiotics; MDR, multi-drug resistant, non-susceptible to at least three classes of antibiotics. Each dot/square/triangle represents the average of three independent experiments for each CF isolate.

**FIGURE 2:**
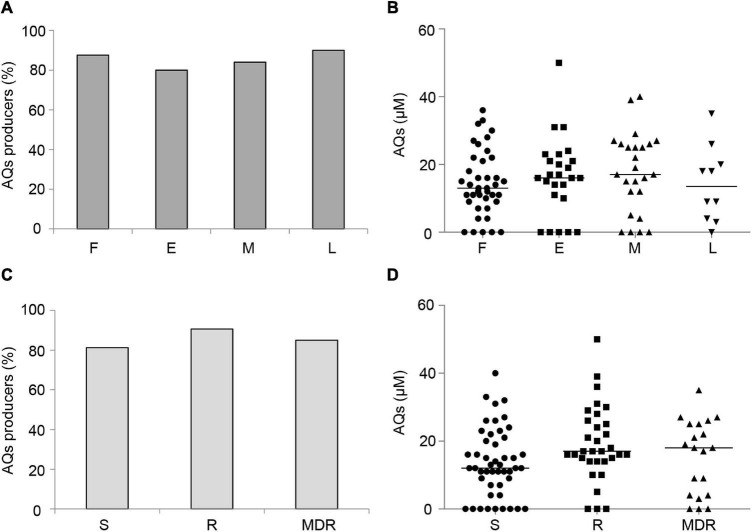
Production of AQs by CF isolates. Percentage of QS signal molecule-producing isolates grouped by duration of the chronic lung infection **(A)** or by antibiotic susceptibility pattern **(C)**. AQs levels produced by CF isolates grouped by duration of the chronic lung infection **(B)** or antibiotic susceptibility pattern **(D)**. Horizontal lines are the median value. Differences between groups are statistically not significant. F, first isolate; E, chronic early; M, chronic middle; L, chronic late; S, susceptible to all antibiotic classes; R, non-susceptible to one or two classes of antibiotics; MDR, multi-drug resistant, non-susceptible to at least three classes of antibiotics. Each dot/square/triangle represents the average of three independent experiments for each CF isolate.

Both the percentage of 3OC_12_-HSL-proficient isolates (Figure 1A) and the level of 3OC_12_-HSL produced by these isolates (Figure 1B) showed an inverse correlation trend with respect to the duration of chronic lung infection. Accordingly, both the percentage of 3OC_12_-HSL-proficient isolates and 3OC_12_-HSL levels were higher in S and R groups with respect to the MDR group (Figures 1C,D), and the latter were more abundant among middle and late CF isolates (Supplementary Table 2). These results are in overall accordance with the existing literature (Hoffman et al., 2009; Bjarnsholt et al., 2010; Jiricny et al., 2014; Feltner et al., 2016; Winstanley et al., 2016). Conversely, both the percentage of AQs proficient isolates and the level of AQs produced by these isolates did not correlate with the stage of infection or the antibiotic resistance profile (Figure 2).

As mentioned in the introduction, in *P. aeruginosa* reference strains (*e.g.*, PAO1 or PA14) the *las* system positively regulates the *pqs* system, hence 3OC_12_-HSL levels correlate with AQs levels (Lee and Zhang, 2015; Papenfort and Bassler, 2016). However, the statistical analysis performed on the 63 CF isolates able to produce both 3OC_12_-HSL and AQs did not highlight a significant correlation between 3OC_12_-HSL and AQs levels produced by each isolate, suggesting a possible rewiring of the QS regulatory cascade in several CF isolates (Supplementary Figure 2).

In order to obtain comprehensive overview of all major QS signal molecules produced by the CF isolates, the ability to produce C_4_-HSL was also assessed. To this aim, a bioluminescent biosensor specific for C_4_-HSL (C4-Rep) was employed in co-cultivation with each CF isolate. Compared to the cell-free supernatants quantitative method used for measuring 3OC_12_-HSL and AQs levels, co-cultivation is more convenient for discriminating between C_4_-HSL producers and non-producers (details in section “Materials and Methods”). The PAO1 mutant Δ*lasI* Δ*rhlI*, unable to produce AHLs, was used as reference control. Briefly, we measured bioluminescence emission in 14 biological replicates of the PAO1 Δ*lasI*Δ*rhlI* and C4-Rep co-culture. The mean of these data was 1819 ± 701.9 RLU/OD_600_. According to the three-sigma rule (Pukelsheim, 1994), all CF isolates showing RLU/OD_600_ < 3924.7 were considered not significantly different from PAO1 Δ*lasI*Δ*rhlI*, hence unable to produce C4-HSL (Supplementary Figure 3).

Results showed that 78 CF isolates produced detectable levels of C_4_-HSL (Supplementary Table 2), and that the number of C_4_-HSL producers was inversely correlated with the duration of chronic lung infection and the antibiotic resistance profile (Supplementary Figure 4). Also these results are in accordance with the existing literature (Bjarnsholt et al., 2010).

**FIGURE 3:**
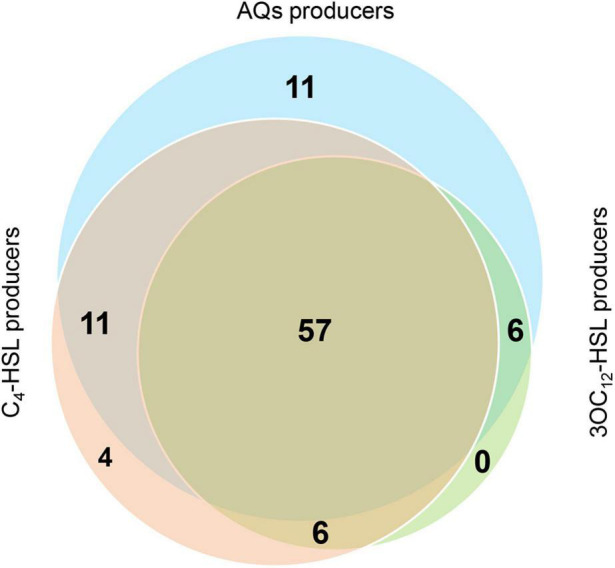
Overview of QS signal molecules produced by CF isolates. Venn diagram showing the CF isolates producing the indicated QS signal molecules.

The overall picture of QS signals production in our collection is that 85 CF isolates produce AQs, 78 isolates produce C_4_-HSL and 69 isolates produce 3OC_12_-HSL. Of these, 57 isolates produce all signal molecules, 23 isolates produce only two of the three signals (C_4_-HSL and AQs, 11 isolates; 3OC_12_-HSL and C_4_-HSL, 6 isolates; 3OC_12_-HSL and AQs, 6 isolates) and 15 isolates produce only one signal (C_4_-HSL, 4 isolates; AQs, 11 isolates). Interestingly, isolates producing exclusively 3OC_12_-HSL were not found in the collection (Figure 3 and Supplementary Table 3).

In conclusion, only 5% of the CF isolates analyzed in this study is impaired in the production of all QS signal molecules, highlighting the importance of at least one QS regulatory pathway in the infection. In addition, *pqs-*proficient CF isolates resulted more frequent than *las-* and *rhl-*proficient isolates, and their frequency did not decrease with the progression of the chronic CF infection.

### Effect of Clofoctol and Niclosamide

**FIGURE 4:**
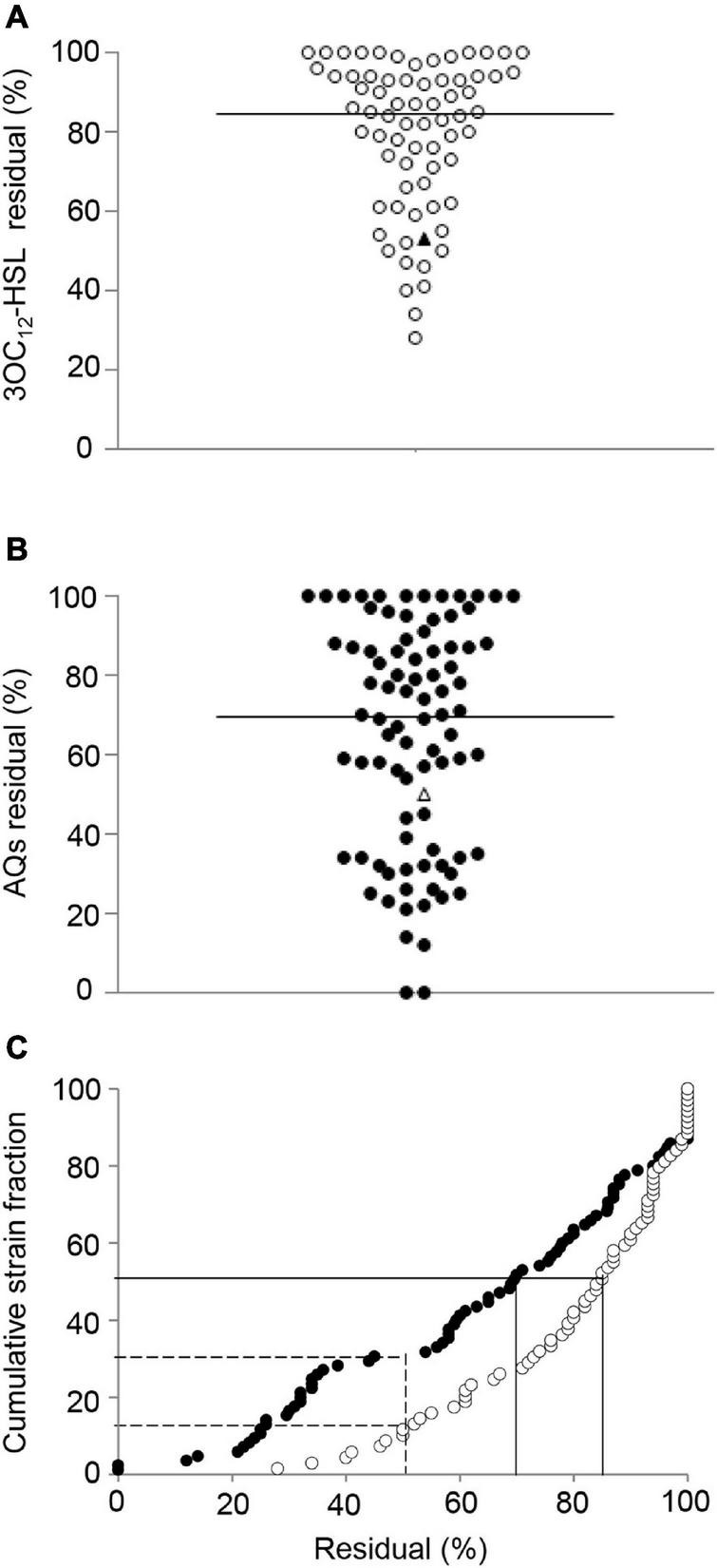
Effect of niclosamide and clofoctol on QS signal molecules produced by CF isolates. Residual levels of 3OC_12_-HSL **(A)** or AQs **(B)** produced by CF isolates grown in the presence of niclosamide or clofoctol, respectively. Residual levels are reported as percentage relative to the untreated samples, considered as 100%. Each dot represents a CF isolate. Triangles represent the laboratory strain PAO1. Horizontal lines are the median value. The average of three independent experiments is reported. The 3OC_12_-HSL and AQs residual levels after niclosamide (white dots) and clofoctol (black dots) treatment are also shown in panel **(C)** reporting their cumulative distribution plot. Continuous lines intercept 3OC_12_-HSL or AQs median residual levels; dashed lines intercept the percentage of the CF isolates showing a residual level ≤ the residual level in PAO1. Average values obtained from three independent experiments are reported.

To investigate to which extent the anti-QS activity formerly documented in reference laboratory strains is conserved in CF isolates, niclosamide and clofoctol were tested as model drugs targeting the *las* and *pqs* QS systems, respectively. Preliminary experiments carried out with a small number of strains showed that 100 μM niclosamide affected the growth of some CF isolates. However, 20 μM niclosamide and 100 μM clofoctol did not affect the grow rate and growth yield of CF isolates and both caused 50% reduction of 3OC_12_-HSL and AQs production, in PAO1 reference strain (Figure 4). Hence, 20 μM niclosamide and 100 μM clofoctol were used to challenge the CF isolates able to produce 3OC_12_-HSL or AQs, respectively.

Figure 4 shows the levels of 3OC_12_-HSL or AQs produced by each isolate after treatment with either niclosamide or clofoctol, expressed as residual levels, *i.e.*, the percentage with respect to the levels of signal molecule produced by the corresponding untreated control. The response of CF isolates to niclosamide treatment ranged from unaffected (100% residual level) to about 20% residual level (Figure 4A). In the case of clofoctol, AQs residual levels ranged from unaffected to total inhibition of AQs production (*i.e.*, undetectable AQs levels in the treated sample) (Figure 4B). Overall, 50% of the isolates treated with niclosamide or clofoctol produced ≤ 85% and ≤ 70% residual levels of signal molecule, respectively. In addition, only 16 and 30% of the CF isolates showed a reduction of the QS-signal levels comparable or lower than that observed for the PAO1 reference strain after treatment with niclosamide or clofoctol, respectively (Figure 4C). Overall, both niclosamide and clofoctol were significantly less effective on CF isolates than on the PAO1 reference strain.

Interestingly, the correlation between 3OC_12_-HSL or AQs levels produced by the untreated sample and the respective residual levels after niclosamide or clofoctol treatment was definitively weak (*r* < 0.1) and statistically insignificant (Supplementary Figures 5A,B). Moreover, by considering CF isolates that produced both 3OC_12_-HSL and AQs, statistical analysis showed that there was no correlation between 3OC_12_-HSL and AQs residual levels after the treatment with niclosamide and clofoctol, respectively (Supplementary Figure 5C). This is in line with the suggestion that the *pqs* system is unlinked from the *las* system in the majority of the CF isolates of the collection.

**FIGURE 5:**
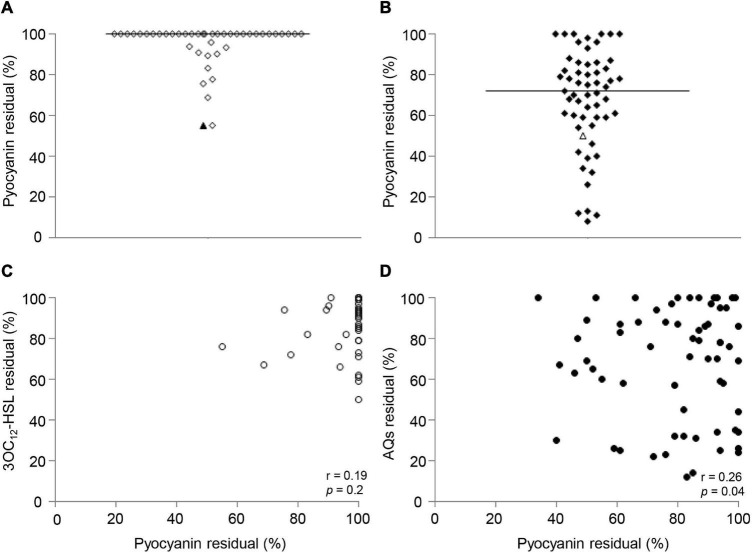
Effect of niclosamide and clofoctol on pyocyanin production and correlation with QS signal levels. Residual levels of pyocyanin in CF isolates grown in presence of niclosamide **(A)** or clofoctol **(B)**, reported as percentage with respect to the relative untreated samples. Each diamond represents an isolate. Triangles represent the laboratory strain PAO1. Black lines represent the median values. **(C)** XY correlation between pyocyanin residual levels and 3OC_12_-HSL residual levels (%) after niclosamide treatment. Each dot represents a CF isolate producing both pyocyanin and 3OC_12_-HSL (*n* = 42), Pearson correlation test: *r* = 0.19; *p* = 0.2. **(D)** XY correlation between pyocyanin residual levels and AQ residual levels (%) after clofoctol treatment. Pearson correlation test: *r* = 0.26; *p* < 0.05. Each dot represents a CF isolate producing both pyocyanin and AQs (*n* = 56). Each dot/diamond/triangle represents the average of three independent experiments for each CF isolate.

Pyocyanin is a major *P. aeruginosa* virulence factor in CF infection (Denning et al., 1998), whose production is positively regulated by both the *las* and the *pqs* systems in reference strains such as PAO1 or PA14 (Jimenez et al., 2012; García-Reyes et al., 2020). Accordingly, both niclosamide (20 μM) and clofoctol (100 μM) decreased pyocyanin production of about 50% in the PAO1 strain (Figures 5A,B). To evaluate if niclosamide and clofoctol antivirulence activity against PAO1 was maintained in CF isolates, their effect on pyocyanin production was determined.

Preliminary analysis showed that 42 out of the 69 3OC_12_-HSL-proficient isolates and 56 out of the 85 AQs-proficient isolates produced detectable levels of pyocyanin. The analysis of the effect of niclosamide in the 42 *las*- and pyocyanin-proficient isolates showed that pyocyanin production was unaffected or only marginally affected by niclosamide in the majority of isolates, with residual pyocyanin levels ≤ 70% observed only for two CF isolates (Figure 5A). Conversely, pyocyanin residual levels in the 56 *pqs*- and pyocyanin-proficient CF isolates treated with clofoctol ranged from unaffected (100% residual level) to about 5%, with pyocyanin residual levels ≤ 70% in about half of the isolates (Figure 5B). In addition, no significant correlation between 3OC_12_-HSL and pyocyanin reduction was observed (Figure 5C), while a weak (*r* = 0.26) but statistically significant (*p-*value = 0.04) correlation between AQs and pyocyanin residual levels was found (Figure 5D).

**FIGURE 6:**
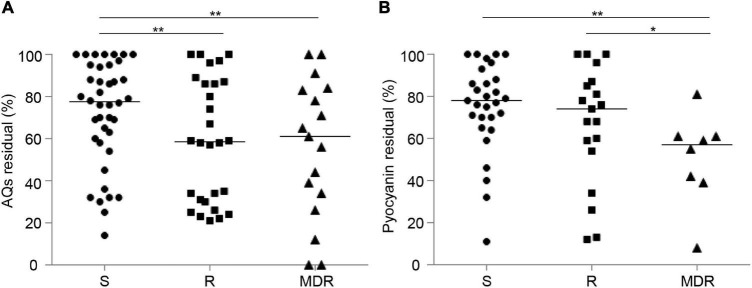
Effect of clofoctol in CF isolates grouped by antibiotic resistance pattern. AQs **(A)** and pyocyanin **(B)** residual levels in CF isolates grown in presence of clofoctol, reported as percentage with respect to the untreated samples considered as 100%. Isolates are clustered by antibiotic susceptibility pattern. S, susceptible to all antibiotic classes; R, non-susceptible to one or two classes of antibiotics; MDR, multi-drug resistant, non-susceptible to at least three classes of antibiotics. Black lines represent the median values. Statistically significant differences among groups are highlighted by asterisks (*p < 0.05; **p < 0.01; KS-test). Each dot/square/triangle represents the average value of three independent experiments for each CF isolate.

The residual levels of 3OC_12_-HSL, AQs or pyocyanin after niclosamide or clofoctol treatment did not appear to be related to the stage of infection (Supplementary Figures 6A,C,E,F), and no correlation was observed between the response to niclosamide and the antibiotic resistance profile (Supplementary Figures 6B,D). However, it can be noticed that the effect of clofoctol on both AQs and pyocyanin production was more significant in CF isolates belonging to the MDR group, compared with isolates belonging to the S group (Figure 6).

The overall scarce effect of niclosamide on pyocyanin production could be explained by a *las*-independent regulation of pyocyanin biosynthetic genes in at least some CF strains. To gain more insights into the antivirulence activity of niclosamide, the effect of this drug was tested on the protease elastase, a *P. aeruginosa* virulence factor strictly controlled by the *las* QS system (Gambello and Iglewski, 1991) and strongly inhibited by niclosamide in the *P. aeruginosa* reference strain PA14 (Imperi et al., 2013). Accordingly, niclosamide inhibited elastase production (about 60% residual levels) in the reference strain PAO1 under our experimental conditions (Supplementary Figure 7). Hence, the effect of niclosamide on elastase production was tested in the 3OC_12_-HSL-producing CF isolates. Preliminary experiments showed that 63 over the 69 3OC_12_-HSL-producing isolates were endowed with detectable elastolytic activity under standard laboratory conditions. However, niclosamide treatment scarcely affected elastase production in the tested CF isolates, with none isolate showing more than 30% reduction of elastase activity after treatment (Supplementary Figure 7).

Taken together, the above results provide evidence that the ability of niclosamide and clofoctol to inhibit the production of QS signal molecules and QS-controlled virulence factors is highly variable and strain-dependent, and suggest that anti-*pqs* drugs might be more effective as antivirulence agents than anti-*las* drugs in CF therapy.

## Discussion

Dozens of molecules targeting the *P. aeruginosa* QS systems and causing a reduction of virulence factors production (*e.g.*, pyocyanin, proteases) *in vitro* have been described, and some of these have been proven to be active in animal models of infection with non-CF *P. aeruginosa* reference strains (*e.g.*, PAO1 or PA14; Rampioni et al., 2014; Soukarieh et al., 2018a). Nevertheless, the frequent isolation of CF isolates with mutations in the *lasR* gene (Smith et al., 2006; Hoffman et al., 2009; Folkesson et al., 2012; Feltner et al., 2016; Winstanley et al., 2016), causing inactivation of the *las* QS system, has been considered a caveat for the development of QS inhibitors in CF therapy (García-Contreras et al., 2013). Beyond mutations in the QS system targeted by the antivirulence drug, a CF isolate could be more resistant to an antivirulence drug than a reference strain for several reasons, including overexpression of efflux pumps or modifications of the cell envelope reducing drug internalization (Maeda et al., 2012; García-Contreras et al., 2013). On these assumptions, this study has investigated QS functionality and susceptibility to anti-QS drugs in a large collection of CF isolates, with the objective of investigating the suitability of QS inhibitors for CF therapy.

Concerning the distribution of isolates able to produce QS signal molecules in our collection, the vast majority (95%) of them produced at least one signal, and 57% all the three *P. aeruginosa* QS signals (Supplementary Table 3). Interestingly, 5 isolates defective in all QS systems were almost equally distributed among the sub-groups defined by stage of infection (Supplementary Tables 2, 3). These results support the importance of QS in *P. aeruginosa* infection, though they also suggest that a limited number of isolates could be able to sustain an intermittent or chronic infection even if defective in all QS systems. Deeper investigations should be conducted to analyze the specific virulence phenotypes of these QS-null isolates.

The *las* signal molecule 3OC_12_-HSL was the QS signal produced by the lowest number of CF isolates (69% proficient isolates), compared with C_4_-HSL (78% proficient isolates) and AQs (85% proficient isolates), likely denoting a hierarchy of QS systems utility in the CF chronic infection. Indeed, no isolate produced exclusively 3OC_12_-HSL, while 4 and 11 isolates of our collection produced only C_4_-HSL and AQs, respectively. In addition, the first isolate group contains about 20% of *las*-defective strains, suggesting that loss-of-function mutations in the *las* QS system can emerge early during infection, and not only occur following long-term adaptation to the chronic CF lung. This is in line with a recent study reporting similar percentages of environmental *P. aeruginosa* strains unable to produce 3OC_12_-HSL (Groleau et al., 2021). Both the levels of 3OC_12_-HSL and the percentage of 3OC_12_-HSL-proficient strains decrease in MDR with respect to R and S isolates, and a similar descending trend is observed along the years of chronic lung infection. Hence, even if *las* defective strains coming from the environment can establish early infections, a trend toward the selection of *las*-defective and MDR strains during the infection is recognizable.

Overall, with respect to the ability to produce *las* and *rhl* QS signal molecules, the CF isolates here investigated have features already reported by others, indicating that the collection used in this study faithfully represents the *P. aeruginosa* strains landscape in CF (Bjarnsholt et al., 2010; Jiricny et al., 2014; Feltner et al., 2016; Winstanley et al., 2016).

Functionality of the *pqs* system in CF isolates has been poorly investigated so far. Here it is shown that 85% of CF isolates produce AQs, hence the *pqs* system appears to be the most functionally conserved QS circuit in *P. aeruginosa* CF isolates. Interestingly, the percentage of *pqs*-proficient isolates and the level of AQs they produce do not vary based on the stage of the infection and on the antibiotic resistance pattern of the CF isolates. This suggests that *pqs* mutations are not positively selected along the years of infection and raises the possibility that anti-*pqs* drugs could be used at any stage of the CF infection.

Our findings also suggest that the hierarchically dominant role of the *las* system over the *rhl* system, typically reported in PAO1 and PA14 model strains, can be lost in CF isolates, as previously documented in clinical and environmental isolates (Feltner et al., 2016; Chen et al., 2019; Cruz et al., 2020; Groleau et al., 2021). Moreover, since no correlation was observed between 3OC_12_-HSL and AQs levels in our CF isolates, it can be hypothesized that also the *pqs* system is independent of the *las* system, at least in our experimental setting. This is an interesting issue that could be further explored in future studies.

Until now, very few studies showed the effect of QS inhibitors on CF clinical isolates (Rampioni et al., 2017b; D’Angelo et al., 2018; Baldelli et al., 2020; Mahan et al., 2020; Papa et al., 2021; Soukarieh et al., 2021), and only in one study the antivirulence activity of a drug targeting the *las* QS system, furanone C-30, was tested in a collection of fifty CF isolates, revealing highly variable response and high frequency of resistance to the QS inhibitor in CF isolates (García-Contreras et al., 2015), in overall accordance with this study.

The ability of niclosamide to reduce 3OC_12_-HSL levels in the *las*-proficient CF isolates was variable, with few isolates showing substantial reduction of this signal molecule. In addition, the 31 isolates unable to produce 3OC_12_-HSL should virtually be considered resistant to any inhibitor targeting the *las* QS system. Therefore, the overall range of efficacy of niclosamide against CF isolates appears very low. Moreover, in the isolates showing reduced 3OC_12_-HSL production in response to niclosamide treatment, a correlation with elastase or pyocyanin reduction was not observed. This is in agreement with the *las*-independent production of virulence factors reported in CF isolates by others (García-Contreras et al., 2015; Feltner et al., 2016; Chen et al., 2019; Cruz et al., 2020; Groleau et al., 2021).

Concerning clofoctol, about 50% of the 85 AQs-producing isolates showed substantial reduction of AQs (≤ 70% of residual activity) when treated with this drug. AQs residual levels in clofoctol-treated isolates were evenly variable, with several isolates showing susceptibility equal or higher than the reference strain PAO1. Similar results were reported in our previous study using a small subset of CF strains (*n* = 20) belonging to the present collection (D’Angelo et al., 2018). In addition, a weak but statistically significant correlation between the reduction of AQs and pyocyanin levels was observed upon clofoctol treatment, suggesting that inhibition of the *pqs* QS system has a positive effect in attenuating the production of virulence factors. Overall, only 15% of CF isolates were *pqs*-deficient, and the majority of *pqs*-proficient isolates were found to be susceptible, to various extent, to clofoctol treatment.

Since in this study different concentrations of niclosamide and clofoctol were used, and their pharmacology profoundly differs, the range and extent of activity of these two drugs cannot be directly compared. However, the results obtained with clofoctol are interesting when considering that for many CF patients the therapeutic choices for controlling *P. aeruginosa* chronic infection could be limited by the multidrug-resistant profile of the infecting strains. In this view, it is worth highlighting that MDR strains are significantly more susceptible to clofoctol than strains sensitive to all antibiotic classes (S) or resistant to one or two different antibiotic classes (R). Interestingly, the few resistance mechanisms described so far for anti-QS drugs consist mainly in increased activity of efflux pumps, the same mechanism that often confers MDR resistance (Maeda et al., 2012; García-Contreras et al., 2013, 2015; García-Contreras, 2016). Since MDR CF strains are more susceptible to clofoctol than S and R strains, clofoctol insensitivity could be mostly mediated by mechanisms different from active efflux of the drug. The clofoctol insensitive CF strains identified in this study could be exploited in the future to investigate the mechanism of resistance to this QS drug.

Overall, this study indicates that, among *P. aeruginosa* QS systems, the *pqs* system should be considered the best target to develop antivirulence drugs for CF therapy. The importance of the *pqs* QS system in *P. aeruginosa* CF infection is also supported by studies showing that AQs levels in the CF sputum correlate with the clinical status of the patient (Barr et al., 2015), and that AQs are suitable biomarkers for culture-independent prediction of *P. aeruginosa* burden in CF adult patients (Zain et al., 2021). Nevertheless, since the *las* QS system is hierarchically dominant over the *rhl* and *pqs* system in *P. aeruginosa* reference strains, the research of QS inhibitors has been targeted mainly toward this system (Rampioni et al., 2014; Soukarieh et al., 2018a). However, several studies reported the identification of molecules targeting the *pqs* system, including: metilantranilate (Calfee et al., 2001); farnesol (Cugini et al., 2007); halogenated anthranilic acid (Lesic et al., 2007); quinazolidine derivatives (Ilangovan et al., 2013; Soukarieh et al., 2018b,^2021^); benzamide-benzimidazole (Starkey et al., 2014; Maura and Rahme, 2017); pimozide (Mellini et al., 2019); nitrofurazone and erythromycin estolate (Baldelli et al., 2020); 2-sufonylpyrimidines (Thomann et al., 2016a,b). Compared with these anti-*pqs* drugs, clofoctol has the advantage of being already used in humans for the treatment of pulmonary infections, hence the delivery of this drug to the CF clinical setting should in principle be more straightforward.

## Data Availability Statement

The original contributions presented in the study are included in the article/Supplementary Material, further inquiries can be directed to the corresponding author.

## Author Contributions

DC, GG, AF, and EF performed the experiments. LL, GR, FI, and PV designed the experiments. CM performed the statistical analysis. EF provided the clinical isolates. LL conceived the study. LL, DC, and GG wrote the manuscript. LL, PV, and FI contributed to the reagents and materials. All authors analyzed the data, corrected and amended the draft of the manuscript and approved the submitted version.

## Conflict of Interest

The authors declare that the research was conducted in the absence of any commercial or financial relationships that could be construed as a potential conflict of interest.

## Publisher’s Note

All claims expressed in this article are solely those of the authors and do not necessarily represent those of their affiliated organizations, or those of the publisher, the editors and the reviewers. Any product that may be evaluated in this article, or claim that may be made by its manufacturer, is not guaranteed or endorsed by the publisher.
