# Supplementary material for: In vitro Activity of Antivirulence Drugs Targeting the las or pqs Quorum Sensing Against Cystic Fibrosis Pseudomonas aeruginosa Isolates
Source: Front Microbiol. 2022 Apr 25;13:845231. doi: 10.3389/fmicb.2022.845231 (PMC9083110; doi:10.3389/fmicb.2022.845231)
Supplement: Supplementary file 1 [file Data_Sheet_1.PDF]

Supplementary Material

***In vitro* activity of antivirulence drugs targeting the *las* or *pqs* quorum sensing against cystic fibrosis *Pseudomonas aeruginosa* isolates**

Diletta Collalto[1†], Giulia Giallonardi[2†], Alessandra Fortuna[1], Carlo Meneghini[1], Ersilia Fiscarelli[3], Paolo Visca[1], Francesco Imperi[1,4], Giordano Rampioni[1,4], Livia Leoni[1]*

[1]Department of Science, University Roma Tre, Rome, Italy; [2]Wellcome Centre for Integrative Parasitology, Institute of Infection, Immunity and Inflammation, University of Glasgow, Glasgow, United Kingdom; [3]Laboratory of Cystic Fibrosis Microbiology, Bambino Gesú Hospital, Rome, Italy; [4]Santa Lucia Foundation (IRCCS), Rome, Italy.

[†] These authors have contributed equally to this work and share first authorship.

*** Correspondence:**
Livia Leoni
livia.leoni@uniroma3.it

1 Supplementary Figures and Tables

1.1 Supplementary Figures

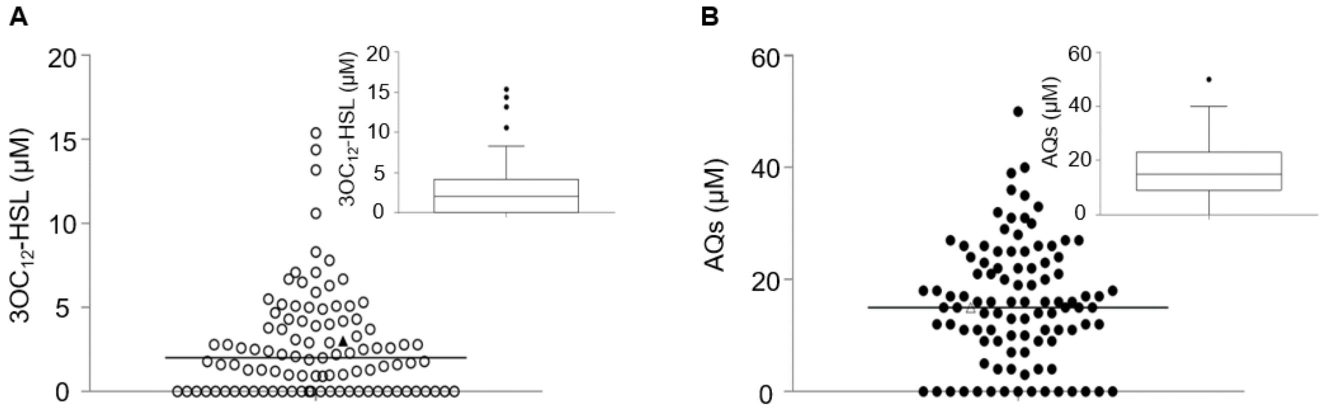

Supplementary Figure 1. Levels of 3OC12-HSL and AQs produced by CF isolates. Levels of 3OC12-HSL **(A)** and AQs **(B)** were measured in the cell-free supernatant of CF isolates. Each dot represents the average of three independent experiments for each CF isolate. Triangles represent the laboratory strain PAO1. The same values are shown at the top of each graph by a box plot with Tukey whiskers, in which the black dots represent the outliers. The black lines represent median values.

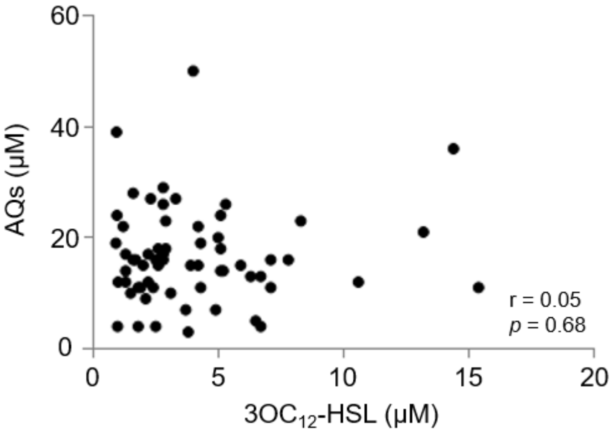

Supplementary Figure 2. Correlation between 3OC12-HSL and AQs levels. XY plot representing on the y-axis AQs levels (µM) and on the x-axis 3OC12-HSL levels (µM) produced by CF isolates. Each dot represents the average of three independent experiments for each CF isolate producing both AQs and 3OC12-HSL (n = 63; Pearson correlation test: r = 0.05, *p* = 0.68).

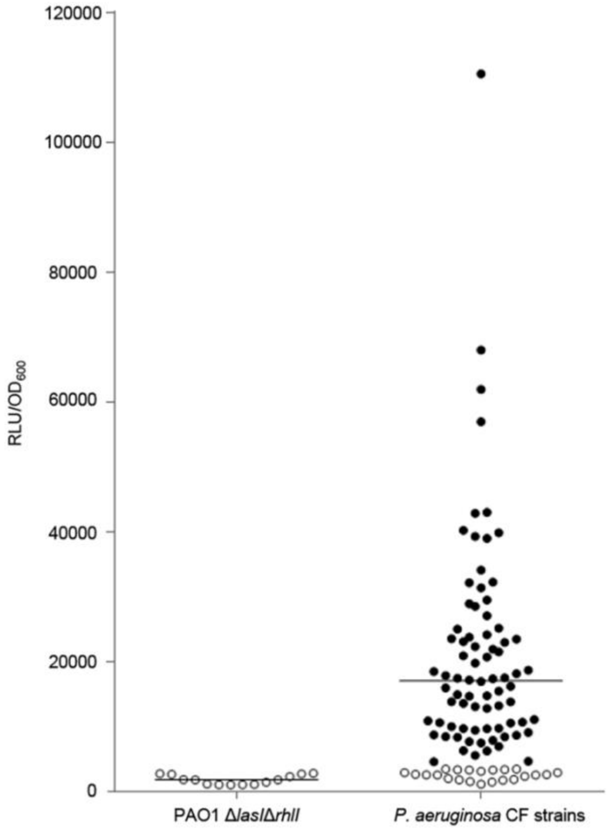

Supplementary Figure 3. Screening of *P. aeruginosa* CF strains to identify the producers of C_4-HSL. Dot plots represent relative light units (RLU) normalized for OD_{600} emitted by the biosensor C4-Rep co-cultured with PAO1 Δ*lasI*Δ*rhlI* strain (14 replicates, left) or each one of the 100 CF isolates (mean of three independent experiments). White dots represent strains producing undetectable levels of C_4-HSL, black dots represent CF strains producing detectable levels of C_4-HSL. The horizontal lines represent mean values.

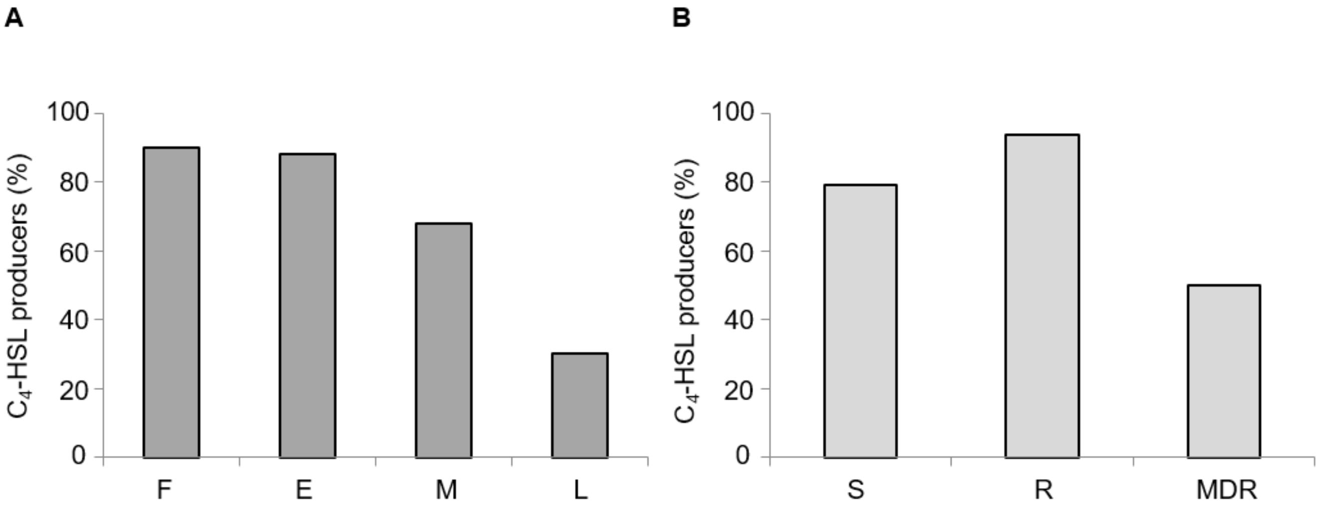

Supplementary Figure 4. C_4-HSL producer isolates grouped by duration of the chronic lung infection, or by antibiotic susceptibility pattern. Each category of isolates grouped by duration of chronic infection **(A)** or antibiotic resistance pattern **(B)** is reported as percentage of strains able to produce C_4-HSL.

F: first isolate; E: chronic early; M: chronic middle; L: chronic late; S: susceptible to all antibiotic classes; R: non-susceptible to one or two classes of antibiotics; MDR: multi-drug resistant, non-susceptible to at least three classes of antibiotics. The average of three independent experiments is reported.

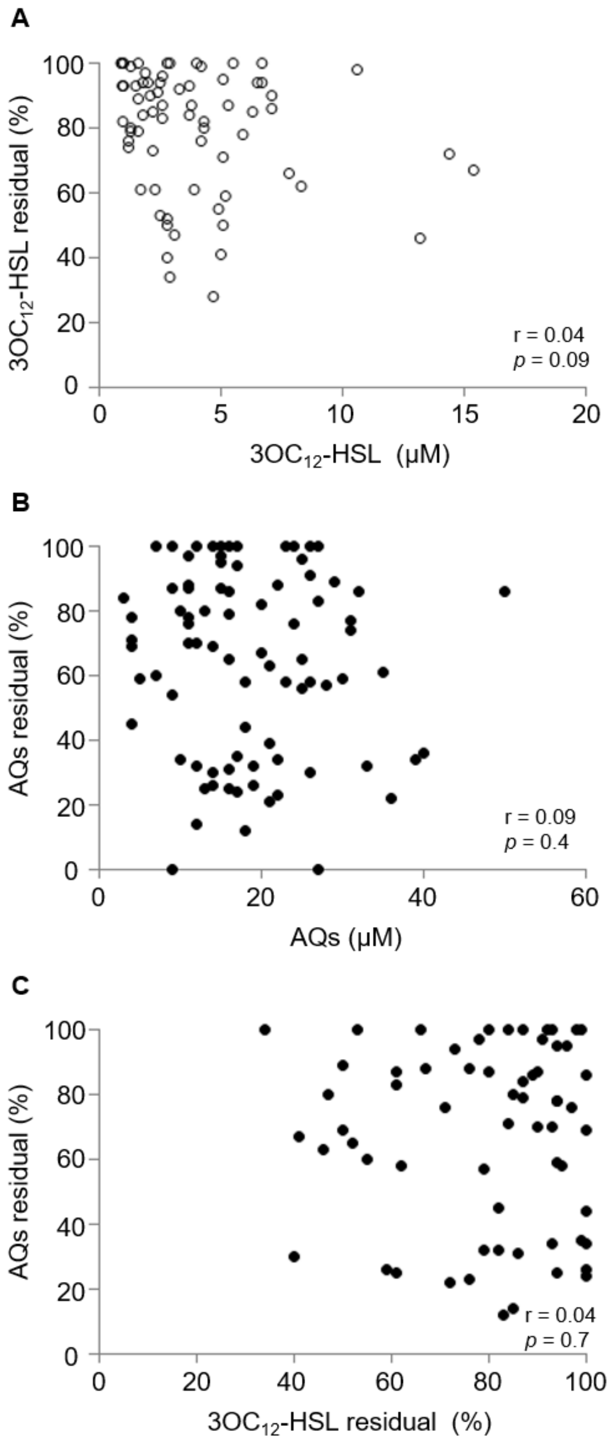

Supplementary Figure 5. Correlation between levels of QS signal produced and anti-QS drug effect. (A) x-axis, $3OC_{12}$-HSL levels (µM); y-axis, $3OC_{12}$-HSL residual levels (%) after niclosamide treatment (Pearson correlation test: r = 0.04 p = 0.09). **(B)** x-axis, AQs levels (µM); y-axis, AQs residual levels (%) after clofoctol treatment (Pearson correlation test: r = 0.09 p = 0.4). **(C)** x-axis

3OC12-HSL residual levels (%) after niclosamide treatment; y-axis, AQs residual levels (%) after clofoctol treatment (Pearson correlation test: r = -0.04 *p* = 0.7).

Each dot represents the average of three independent experiments for each CF strain (A, n = 69; B, n = 85; C, n=63).

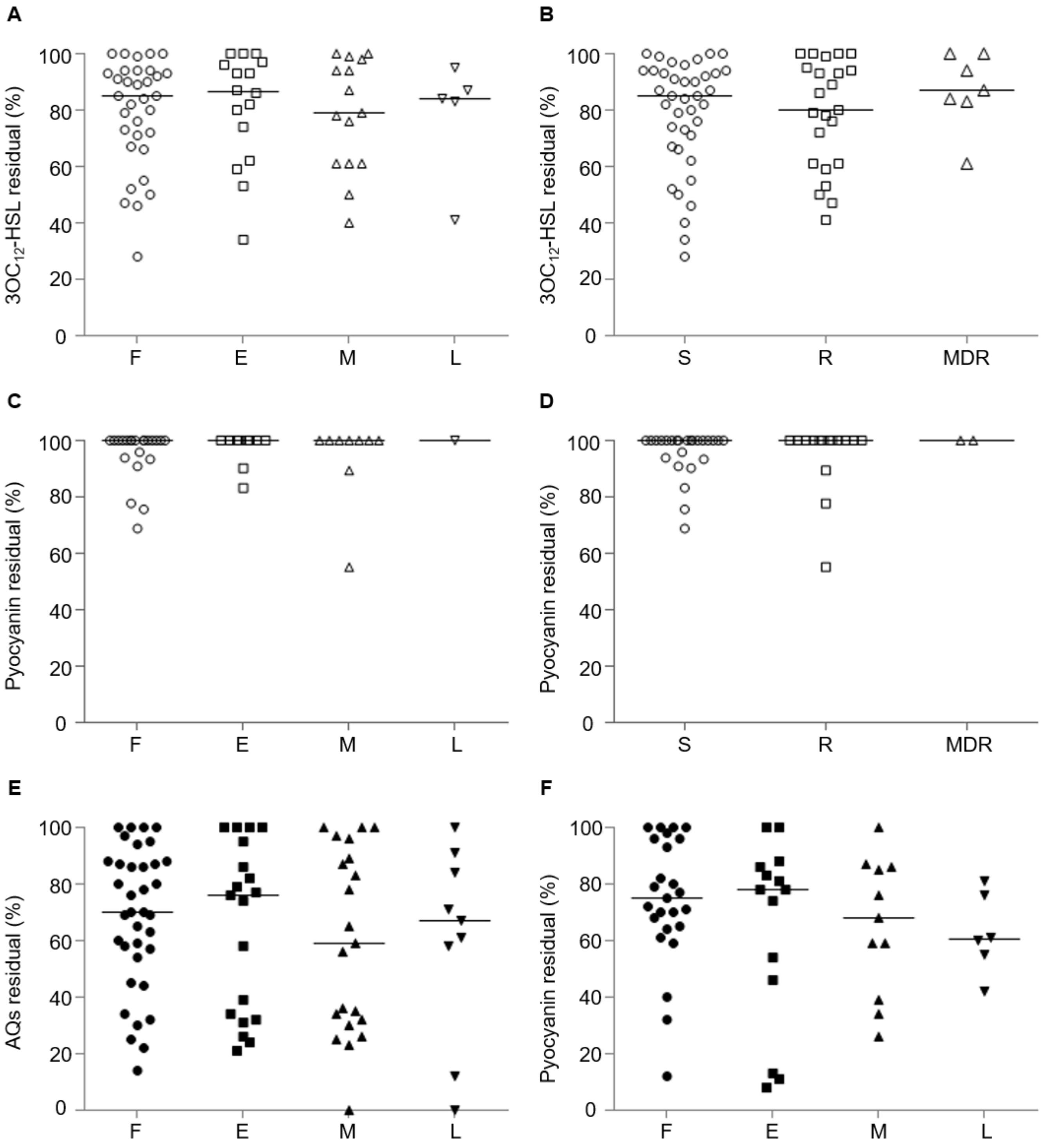

Supplementary Figure 6. QS signals and pyocyanin residual levels after anti-QS treatment, grouped by duration of chronic lung infection, or by antibiotic susceptibility pattern. $3OC_{12}$-HSL and pyocyanin residual levels after niclosamide treatment clustered by **(A, C)** duration of the chronic lung infection and **(B, D)** antibiotic susceptibility pattern. **(E)** AQs and **(F)** pyocyanin

residual levels in CF isolates after clofoctol treatment, clustered by duration of the chronic lung infection.

F: first isolate; E: chronic early; M: chronic middle; L: chronic late; S: susceptible to all antibiotic classes; R: non-susceptible to one or two classes of antibiotics; MDR: multi-drug resistant, non-susceptible to at least three classes of antibiotics. Each dot, square or triangle represents the average of three independent experiments for each CF strain. Black lines represent the median values. Differences among groups are statistically not significant (KS-test).

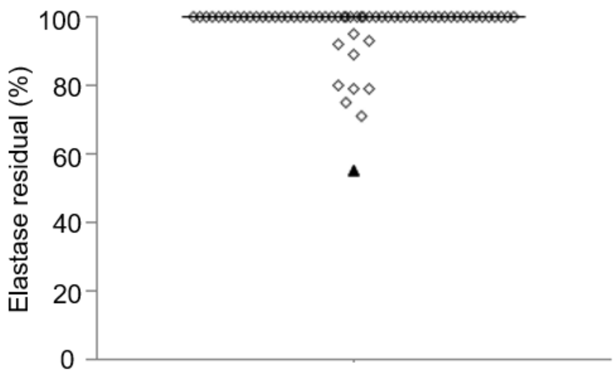

Supplementary Figure 7. Elastase residual levels after niclosamide treatment. Residual levels of elastase activity in CF isolates grown after niclosamide treatment. Each diamond represents the average of three independent experiments for each CF isolate. The black triangle represents the laboratory strain PAO1. Black line represents the median value.

1.2 Supplementary Tables

Supplementary Table 1. Bacterial strains used in this study.

Strain	Relevant characteristics	Reference/Source
Pseudomonas aeruginosa		
PA14		
Δ*lasI* P*rsaL*::*lux* (PA14-R3, $3OC_{12}$-Rep)	PA14 derivative strain deleted in the *lasI* gene carrying chromosomal insertion of the P*rsaL*::*lux* transcriptional fusion; used for the detection of *N*-3-oxododecanoyl-L-homoserine lactone signal molecule.	Massai *et al.*, 2011
PAO1		
wild type	Wild type strain	ATCC 15692
Δ*rhlI* P*rhlA*::*lux* (C_4-Rep)	PAO1 derivative strain deleted in the *rhlI* gene carrying the pMS402 plasmid containing the P*rhlA*::*lux* transcriptional fusion; used for the detection of *N*-butanoyl-homoserine lactone signal molecule; Km^R.	Duan and Surette, 2007
Δ*pqsA* P*pqsA*::*lux* (AQ-Rep)	PAO1 derivative strain deleted in the *pqsA* gene carrying chromosomal insertion of the P*pqsA*::*lux* transcriptional fusion; used for the detection of 2-alkyl-4-quinolones (AQs) signal molecules.	Diggle *et al.*, 2007
Δ*lasI* Δ*rhlI*	PAO1 derivative strain deleted in the *lasI* and *rhlI* genes. It does not produce $3OC_{12}$-HSL and C_4-HSL.	This study[a]

[a] The strain PAO1 Δ*lasI*Δ*rhlI* was generated by standard stepwise allelic exchange using derivatives of the pDM4 plasmid (Milton *et al*., 1996) named pDM4Δ*lasI* and pDM4Δ*rhlI*. For pDM4Δ*lasI* and pDM4Δ*rhlI* construction, DNA fragments of 500 bp, located upstream and downstream of the gene *lasI* and of the gene *rhlI* were amplified from *P. aeruginosa* PAO1 genome by PCR. Each pair of upstream and downstream

sequences was cloned in pDM4 by Xbal/XhoI restriction. Subsequent steps to select and confirm the deletions were carried out as previously described (Fortuna *et al*., 2021)

Supplementary Table 2. Features of the CF strains analyzed in this study.

Colonization[a]	Isolate name[b]	Antibiotic susceptibility[c,d]	$3OC_{12}$-HSL levels (µM)	AQs levels (µM)	Residual $3OC_{12}$-HSL production[e]	Residual AQs production[f]	C_4-HSL production[g]
First isolate	BG1	R	np	14	/	30	yes
	BG4	R	14.4	36	72	22	yes
	BG7	S	6.7	13	94	25	yes
	BG10	S	2.2	12	85	14	yes
	BG13	MDR	2.9	18	100	44	yes
	BG16	S	1.8	11	94	78	yes
	BG19	R	np	30	/	59	yes
	BG22	S	3.7	np	93	/	yes
	BG24	MDR	np	22	/	34	yes
	BG27	S	7.1	11	90	70	yes
	BG30	S	np	33	/	32	np
	BG33	R	1.6	np	100	/	yes
	BG35	S	5.1	24	71	76	yes
	BG37	R	np	26	/	58	yes
	BG39	S	13.2	21	46	63	yes
	BG41	S	np	np	/	/	np
	BG43	S	2.8	16	52	65	yes
	BG45	S	3.3	27	92	100	yes
	BG47	R	3.1	10	47	80	yes
	BG48	S	2	15	94	95	yes
	BG50	S	0.99	12	93	70	yes
	BG52	S	6.3	13	85	80	yes
	BG54	S	2.1	9	90	87	yes
	BG56	R	1.6	28	79	57	yes
	BG57	R	1.6	16	89	86	yes

	BG59	S	4.2	15	99	100	yes
	BG60	S	3.7	7	84	100	yes
	BG62	S	4.2	22	76	88	yes
	BG64	S	4.7	np	28	/	yes
	BG65	S	np	32	/	86	np
	BG66	S	5.5	np	100	/	yes
	BG67	S	6.7	4	100	69	yes
	BG68	S	7.8	16	66	100	yes
	BG69	S	0.97	4	82	45	yes
	BG70	S	4.3	11	80	87	yes
	BG71	S	np	9	/	54	np
	BG72	S	4.9	7	55	60	yes
	BG73	S	5.1	14	50	69	yes
	BG74	S	15.4	11	67	88	yes
	BG75	S	2.4	11	91	97	yes
**Chronic early**	BG2	R	np	21	/	21	yes
	BG5	R	7.1	16	86	31	yes
	BG8	R	5.2	14	59	26	yes
	BG11	R	1.5	10	93	34	yes
	BG14	MDR	np	21	/	39	yes
	BG17	R	2.8	17	100	24	yes
	BG20	R	np	np	/	/	yes
	BG23	S	2.9	23	34	100	yes
	BG25	MDR	np	np	/	/	yes
	BG28	S	2.6	15	96	95	yes
	BG31	S	1	np	100	/	yes
	BG34	R	4	50	100	86	yes

	BG36	S	4.3	19	82	32	yes
	BG38	R	np	31	/	74	yes
	BG40	R	0.95	24	93	100	np
	BG42	R	2.2	17	73	94	yes
	BG44	R	2.5	16	53	100	yes
	BG46	S	8.3	23	62	58	yes
	BG49	S	1.9	11	97	76	yes
	BG51	R	1.3	14	80	100	yes
	BG53	S	2.6	16	87	79	yes
	BG55	S	1.2	np	74	/	yes
	BG58	S	np	np	/	/	np
	BG61	S	np	20	/	82	yes
	BG63	S	np	31	/	77	np
**Chronic middle**	BG3	MDR	np	17	/	100	yes
	BG6	R	1.3	17	99	35	yes
	BG9	S	5.3	26	87	100	yes
	BG12	S	1.3	12	79	32	yes
	BG15	MDR	2.5	4	94	78	yes
	BG18	R	1.7	16	61	25	yes
	BG21	R	np	25	/	96	yes
	BG26	XDR	np	np	/	/	yes
	BG29	R	6.5	5	94	59	yes
	BG32	S	np	np	/	/	yes
	BG76	R	1.2	22	76	23	yes
	BG77	XDR	np	27	/	0	yes
	BG78	MDR	0.91	19	100	26	np
	BG79	R	np	np	/	/	np
	BG80	R	3.9	15	61	87	yes

	BG81	S	2.8	26	40	30	np
	BG82	MDR	2.3	27	61	83	yes
	BG83	R	0.93	39	100	34	yes
	BG84	R	2.8	29	50	89	yes
	BG85	R	5.9	15	78	97	yes
	BG86	S	10.6	12	98	100	np
	BG87	S	np	np	/	/	np
	BG88	MDR	np	25	/	56	np
	BG89	MDR	np	25	/	65	np
	BG90	S	np	40	/	36	np
**Chronic late**	BG91	XDR	np	9	/	0	np
	BG92	XDR	2.6	18	83	12	np
	BG93	MDR	3.8	3	87	84	yes
	BG94	XDR	np	9	/	100	np
	BG95	MDR	np	26	/	91	np
	BG96	R	5.1	18	95	58	yes
	BG97	XDR	1.8	4	84	71	np
	BG98	MDR	np	35	/	61	np
	BG99	MDR	np	np	/	/	np
	BG100	R	5	20	41	67	yes

[a] CF isolates are grouped according to the year of infection in the lung of individuals with cystic fibrosis: first isolate; chronic early (from 2 to 3 years); chronic middle (from 5 to 7 years); chronic late (equal or more than 15 years).

[b] strain name Bambino Gesú hospital, Rome, Italy

[c]MDR, resistant to one or more antibiotics belonging to at least three different classes (highlighted in yellow); S, susceptible to all classes of antibiotics (highlighted in light blue); R, resistant to one or more antibiotics belonging to one or two different classes (highlighted in pink) (European Centre for Diseases Prevention and Control (ECDC), http://ecdc.europa.eu/en/Pages/home.aspx).

[d]A detailed list of the antibiotics to which each isolate is resistant or susceptible is available in (Imperi *et al*., 2019).

[e]Residual production of $3OC_{12}$-HSL in samples treated with 20 μM niclosamide relative to untreated samples, considered as 100%. np, strains that do not produce detectable levels of 3OC12-HSL.

[f]Residual production of AQs in samples treated with 100 μM clofoctol relative to untreated samples, considered as 100%. np, strains that do not produce detectable levels of AQs.

[g]Yes, ability to produce detectable levels of C_4-HSL. np, strains that do not produce detectable levels of C_4-HSL.

Supplementary Table 3. *P. aeruginosa* CF isolates grouped for their ability to produce QS signal molecules.

QS signal(s)	Number of strains	Strain names
$3OC_{12}$-HSL	0	none
C_4-HSL	4	BG20; BG25; BG26; BG32.
AQs	11	BG30; BG63; BG65; BG71; BG88; BG89; BG90; BG91; BG94; BG95; BG98.
$3OC_{12}$-HSL<br>C_4-HSL	6	BG22; BG31; BG33; BG55; BG64; BG66.
$3OC_{12}$-HSL<br>AQs	6	BG40; BG78; BG81; BG86; BG92; BG97.
C_4-HSL<br>AQs	11	BG1; BG2; BG3; BG14; BG19; BG21; BG24; BG37; BG38; BG61; BG77.
$3OC_{12}$-HSL<br>C_4-HSL<br>AQs	57	BG4; BG5; BG6; BG7; BG8; BG9; BG10; BG11; BG12; BG13; BG14; BG15; BG16; BG17; BG18; BG23; BG27; BG28; BG29; BG34; BG35; BG36; BG39; BG42; BG43; BG44; BG45; BG46; BG47; BG48; BG49; BG50; BG51; BG52; BG53; BG54; BG56; BG57; BG59; BG60; BG62; BG67; BG68; BG69; BG70; BG72; BG73; BG74; BG75; BG76; BG80; BG82; BG83; BG84; BG85; BG93; BG96; BG100.
No QS signals	5	BG41; BG58; BG79; BG87; BG99.

References (additional to those included in the main text)

- Duan, K., and Surette, M. G. (2007). Environmental regulation of *Pseudomonas aeruginosa* PAO1 Las and Rhl quorum-sensing systems. *J. Bacteriol.* 189, 4827–4836. doi:10.1128/JB.00043-07.
- Diggle, S. P., Matthijs, S., Wright, V. J., Fletcher. M. P., Chhabra, S. R., Lamont, I. L., et al. (2007). The *Pseudomonas aeruginosa* 4-quinolone signal molecules HHQ and PQS play multifunctional roles in quorum sensing and iron entrapment. *Chem. Biol.* 14, 87–96. doi:10.1016/j.chembiol.2006.11.014.

- Fortuna, A., Bähre, H., Visca, P., Rampioni, G., and Leoni, L. (2021). The two *Pseudomonas aeruginosa* DksA stringent response proteins are largely interchangeable at the whole transcriptome level and in the control of virulence-related traits. *Environ. Microbiol.* 23, 5487–5504. doi:10.1111/1462-2920.15693.
- Milton, D.L., O'Toole, R., Horstedt, P., and Wolf-Watz, H. (1996). Flagellin A is essential for the virulence of *Vibrio anguillarum*. *J. Bacteriol*, 178:1310–1319. doi: 10.1128/jb.178.5.1310-1319.1996.
